# A Study Protocol for an Open-Label Feasibility Treatment Trial of Visual Snow Syndrome With Transcranial Magnetic Stimulation

**DOI:** 10.3389/fneur.2021.724081

**Published:** 2021-09-24

**Authors:** Marissa Grande, Lucas Lattanzio, Isabelle Buard, Allison M. McKendrick, Yu Man Chan, Victoria S. Pelak

**Affiliations:** ^1^Department of Neurology, University of Colorado, Aurora, CO, United States; ^2^Department of Optometry and Vision Sciences, University of Melbourne, Melbourne, VIC, Australia; ^3^Department of Ophthalmology, University of Colorado, Aurora, CO, United States

**Keywords:** visual snow, transcranial magnetic stimulation, open-label treatment trial, visual psychophysics, migraine

## Abstract

**Background:** Visual Snow (VS) syndrome is believed to be due to aberrant central visual processing. Positron Emission Tomography (PET) brain imaging and visual evoked potential studies provide evidence for excessive neuronal activity in the medial temporal lobe, specifically the lingual gyrus, and suggest the VS syndrome is a hyperexcitability syndrome. These data provide the basis for consideration of repetitive transcranial magnetic stimulation (rTMS) as a potential treatment for the VS syndrome.

**Objective:** To publish the study protocol for a pilot study underway at the University of Colorado School of Medicine to investigate the use of rTMS intervention to improve symptoms and visual dysfunction associated with VS. The study aims to determine the adverse events and drop-out rate, evaluate performance of outcome measures, including a novel VS symptom scale, and describe changes in outcomes associated with treatment.

**Methods and Design:** Up to 10 participants meeting criteria for VS syndrome, age 19–65 years, will undergo an open-label intervention consisting of 10 rTMS sessions, occurring 5 days a week over a 2-week period. Participants will complete pre-treatment and post-treatment assessments that include: the Colorado Visual Snow Scale (CVSS), the National Eye Institute Visual Functional Questionnaire—25 (VFQ-25), the General Anxiety Disorder—7 scale (GAD-7), and three psychophysical visual processing tasks.

**Discussion:** Knowledge gained from this pilot study will inform future study planning and provide valuable lessons for future investigation of rTMS for the VS syndrome. An overview of study proceedings thus far demonstrates recruitment challenges associated with the COVID-19 pandemic, and additional challenges that are unique to the VS syndrome and to treatment schedules associated with TMS.

**Registration:** This study has been approved by the Colorado Multiple Institutional Review Board. ClinicalTrials.gov Identifier: NCT04925232.

## Introduction

People with Visual Snow (VS) syndrome perceive small, moving dots, which resemble the TV static of a poorly tuned analog television, in a constant manner throughout their visual field. Other symptoms include palinopsia or visual trails, light sensitivity, excessive awareness of flashes of lights and floaters, tinnitus, and balance problems. VS most often affects young people in the third decade of life, but it can strike anyone at any age, including children and older adults (1, 2). Comorbidity includes migraine headaches, which are present in 60–80% of people with VS (2). There is no effective treatment, and the cause is unknown. Many patients with VS have difficulty with visual functions and can struggle to continue working because of the visual symptoms associated with the syndrome (2). The International Headache Society (IHS) published criteria (3) for the VS syndrome and these criteria are summarized in Table 1.

**Table 1:** Summary of international headache society criteria for visual snow (3).

**Criteria A through D must be met**
A. Dynamic, continuous tiny dots in the entire visual field >3 months
B. At least two of four additional symptoms 1. Palinopsia (visual after-images, trailing of moving objects, or both) 2. Enhanced entoptic phenomena with at least one of the following: excessive floaters in both eyes, excessive blue field entoptic phenomenon, self-lighting perceived with eyes closed, or spontaneous photopsia. 3. Photophobia 4. Nyctalopia
C. Symptoms not consistent with typical migraine visual aura per International Headache Society criteria.
D. Symptoms are not better explained by another disorder (including normal ophthalmic tests and no intake of psychotropic drugs).

The objective of this manuscript is to describe the research protocol for an ongoing, open-label treatment study of repetitive transcranial magnetic stimulation (rTMS) for VS syndrome at the University of Colorado School of Medicine. The aim of this pilot study is to investigate the feasibility of a future randomized controlled trial of rTMS to improve visual function and symptoms associated with VS. Specifically, this study aims to determine: (1) adverse events and drop-out rate, (2) the standard deviation and test-retest reliability of a novel scale (Colorado Visual Snow Scale or CVSS) and performance of three psychophysical visual processing tasks previously investigated by McKendrick et al., and (3) describe changes in outcome measures (described below) following treatment with rTMS.

VS syndrome is believed to be due to aberrant central visual processing that results in excessive neuronal activity in regions of the brain that perform higher order visual processing (4). Given all findings and symptoms, some authors have posited that VS syndrome is due to thalamocortical dysrhythmia (1). Data from Positron Emission Tomography (PET) brain imaging studies and visual evoked potential studies indicate that the excessive neuronal activity occurs in the medial temporal lobes, specifically the right lingual gyrus (5). Although this evidence suggests that the lingual gyrus plays a role in VS, it is not clear whether increased metabolic activity in this region occurs as a result of upstream neuronal dysfunction or is the primary cause of VS syndrome. In either case, similar syndromes with evidence for central nervous system hyperexcitability have the potential to be treated using rTMS, such as cerebellar hyperexcitability (6), central pain syndrome (7), and certain migraine syndromes (8), with each showing modest treatment responses to rTMS. In 2018, the United States Food and Drug Administration (FDA) approved marketing of TMS for the treatment of major depressive disorder, and in 2013, the FDA did the same for certain migraine headache types (9).

Pharmaceutical interventions with anti-epileptics, migraine therapies, and acetazolamide have historically been used to treat VS, and anecdotal evidence and/or limited treatment trials have shown very limited to no efficacy with side effects that often outweigh the benefits [for review of recent treatment data, see (4)]. Consequently, people with VS syndrome can suffer from decreased ability to read, to use a computer, or to drive, and they frequently report poor quality of life due to VS symptoms and anxiety associated with the syndrome (4). Transcranial magnetic stimulation (TMS) utilizes a non-invasive magnetic field to induce electrical currents that are directed at the cerebral cortex discretely, in order to alter neuronal firing. The rTMS method involves the use of continuous “trains” of stimulation for a specified duration of time, in order to produce lasting effects on brain function by either selectively increasing or decreasing neuronal firing. Repetitive TMS has improved outcomes in several neurologic and psychiatric disorders, including chronic tinnitus syndromes without hearing loss (10), which can, on some level, be considered analogous to the disorder of VS. Furthermore, many patients with VS suffer from chronic tinnitus (1, 2).

The goal of rTMS using low frequency (i.e., 1 Hz) stimulation is to decrease neuronal firing, and the inhibitory modulation likely occurs at the level of the synapse, although additional understanding is needed (11). A single pulse of TMS can alter neuronal firing at the moment the pulse is delivered. However, for longer-lasting effects that go beyond the moment of stimulation, repetitive stimulation with 5–20 daily sessions are typically necessary for longer-lasting effects, and 10 sessions have been found to be effective in a variety of disorders [see Table 2 and (12)].

**Table 2:** Summary of data reviewed by Lefaucheur et al. (12).

**Summary of the tables within Lefaucheur et al. (12)**	**Range of pulses per session**	**Range of sessions (one session per 24 h unless noted)**	**Level of evidence (level 1: randomized sham trial through level 4: case series or uncontrolled)**
Table 1. HF-rTMS of M1 contralateral to pain region in neuropathic pain.	1,500–3,000	3–10	2
Table 2. HF-rTMS of bilateral M1 regions in Parkinson's disease (motor symptoms).	600–1,000	5–10	2
Table 3. LF-rTMS of contralesional M1 in motor stroke at the postacute stage.	900–1,800	5–15	2–3
Table 4. HF-rTMS of ipsilesional M1 in motor stroke at the postacute stage.	500–1,350	5–10	2
Table 5. iTBS of ipsilesional M1 in motor stroke at the chronic stage.	600–1,200	10	2, 3
Table 6. HF-rTMS of ipsilesional M1 in post-stroke dysphagia.	500–3,000	5–10	2, 3
Table 7. LF-rTMS of right IFG in post-stroke non-fluent aphasia at chronic stage.	600–1,200	10–20	2, 3
Table 8. rTMS (cTBS) studies in hemispatial neglect (target: left posterior parietal cortex).	4cTBS trains of 15–45 min	2–14	2, 3
Table 9. iTBS of M1 in multiple sclerosis.	600–1,200	10	2
Table 10. LF-rTMS of the auditory cortex in chronic tinnitus.	900–2,000	10 with some mixed (e.g., 4 sessions x 1,800 and 5 sessions x 1,200 pulses)	1, 2, 3
Table 11. LF-rTMS of the auditory cortex combined with HF-rTMS of the left DLPFC in chronic tinnitus.	1,000–2,000	5–10	1, 2
Table 12. HF-rTMS of the left DLPFC in major depressive disorder.	1,600–2,100	10–20 (one study with two sessions in 1 day)	1, 2, 3
Table 13. Deep HF-rTMS of the left DLPFC in major depressive disorder.	1,980–6,012	20	1, 2
Table 14. cTBS/iTBS of the right/left DLPFC in major depressive disorder.	600–1,800	10–30	2, 3
Table 15. LF-rTMS of the left TPC in auditory hallucinations (schizophrenia).	1,000–1,200	4 (two sessions per day)	2
Table 16. HF-rTMS of the left TPJ in auditory hallucinations (schizophrenia).	2,600	4 (two sessions per day)	2
Table 17. HF-rTMS studies of the left DLPFC in negative symptoms of schizophrenia.	1,000–1,500	10–15	1, 2, 3
Table 18. LF-rTMS of the DLPFC in obsessive compulsive disorder.	1,200–2,000	10–15	2, 3
Table 19. Bilateral LF-rTMS of the pre-SMA in obsessive compulsive disorder.	1,200–1,500	18–25	2, 3

*LF, Low Frequency; HF, High Frequency. cTBS, continuous patterned rTMS; iTBS, intermittent patterned rTMS brain stimulation; Note: this VS study protocol uses LF-rTMS, which depresses hyperexcitable neurons, which HF-rTMS activates neuronal activity*.

The mechanism causing more persistent change in neuronal activity is thought to be due to “weakening” of synaptic connections and synaptic plasticity that follows multiple sessions, but the mechanism is not fully understood (11). One theory posits persistent post-synaptic change (i.e., remodeling of the post-synaptic receptor) takes place only after multiple rTMS sessions with many stimulations given per session. These changes at the synaptic level appear to be akin to a physiologic process called long-term depression, or LTD, which can reduce cortical excitability and contribute to cortical plasticity and to learning and memory (13).

Evidence-based guidelines and therapeutic approaches for the use of rTMS in various conditions have been published and recently updated by Lefaucheur et al. in February 10 (12). Those reviewed in detail by Lefaucheur et al. are summarized in Table 2.

To date, there is no published data regarding the use of TMS for the treatment of VS. The goals for publishing the protocol for the ongoing study are to stimulate interest and to share approaches with the scientific community, as well as review the challenges encountered thus far. The methods and the TMS protocol are reviewed, followed by discussion of recruitment during COVID-19, as well as the challenges encountered and potential solutions that could inform planning for future rTMS treatment trials for VS.

## Methods and Analysis

The ongoing study is an open-label feasibility treatment study of VS utilizing a rTMS paradigm. In summary, participants undergo a 2-week treatment intervention for a total of 10 sessions that are ~1 h in duration per session and occur 5 days per week for two consecutive weeks. Assessments described are given at baseline (pre-treatment), post-treatment, and again at 1 and 3 months following treatment.

### Description of Population Being Enrolled

Up to 10 participants ranging in age from 19 to 65 with a diagnosis of VS that meets the International Headache Society (IHS) criteria are being recruited.

Inclusion Criteria:

- Age 19–65 years with a diagnosis of VS that meets IHS criteria
- Able to provide informed consent
- Visual snow must be present for 3 months or more and symptoms must be persistent (i.e., continuous)
- A prior brain magnetic resonance imaging (MRI) scan with and without contrast completed in the past 3 years that does not show signs of clinically significant brain lesion(s) (e.g., no evidence of multiple sclerosis, stroke, brain tumor, cortical heterotopia or other cortical developmental abnormalities, arteriovenous malformation, etc.).

Exclusion Criteria based on TMS safety guidelines (14–16):

- Syndrome meeting criteria for Hallucinogen-persisting perception disorder
- Prior treatment with TMS for any disorder
- Epilepsy, family history of epilepsy, or personal history of seizures
- Any medical condition or medication that increases the risk of seizures
- Pacemaker or another implantable medical device
- Metal in the skull, not including the mouth
- Unstable cardiac, pulmonary, or other systemic illness
- Pregnancy
- Bipolar disorder
- History of suicidality.

### Outcome Measures

#### Questionnaires and Scales

Currently, no outcome measures or scales exist specifically for VS symptoms. For this reason, the CVSS was developed for this study, and is available as Supplementary Material. Additionally, two previously validated scales are being used: The National Eye Institute Visual Functioning Questionnaire—25 (VFQ-25) and the General Anxiety Disorder—7 (GAD-7) scale.

#### Psychophysical Visual Processing Tasks

Recently, McKendrick et al. (17) investigated psychophysical behavioral measures in people with VS compared to controls and found that VS participants showed statistically significant reduced center-surround contrast suppression and elevated luminance increment threshold detection in noise. These findings are consistent with the theory of cortical hyperexcitability. These tasks are, therefore, being employed in this feasibility study to investigate their use as potential markers of treatment efficacy.

The tasks detect extensively studied physiological properties of the visual system that have been used to explore the “balance between inhibition and excitation” and are described elsewhere (17, 18). In brief, for the center-surround matching task, observers are asked to compare the contrast between two small striped patches that are presented side-by-side. The “reference patch” is 40% contrast and is surround by a larger annulus of 95% contrast. The variable contrast small “target patch” is presented alone. Using a spatial forced-choice paradigm, participants must choose which patch is perceived as higher contrast. The strength of the influence of the surround annulus on the perception of the central patch contrast is a measure of the degree of center-surround suppression for each observer. The higher contrast surround (i.e., annulus) should suppress the perceived contrast of the central patch. The magnitude of this suppression of perceived contrast has been noted to be reduced in people with VS (17). For the luminance noise task, two squares filled with luminance noise are presented side-by-side and an observer must report which of the two stimuli also contains a circular luminance increment. Both high noise and low noise squares are used, and the luminance detection threshold is determined for each observer for each noise level. As noted, those with VS have been found to have a higher luminance detection threshold for luminance increments presented on both low and higher pixelated noise backgrounds (17). Learning effects were examined by McKendrick et al. (18) and were not found. The third task measures the ability to determine the global motion direction of a briefly presented field of moving dots presented within a circular window. Within the dot motion movie, some of the dots move in a coherent direction (either left or right, selected at random on each trial), while the remaining dots move in random directions (noise dots). On each trial, the observer indicates the perceived direction of global motion, with the threshold being measured as percent coherence (the percentage of dots in the pattern moving in the signal direction to correctly perceive the direction of motion). Full details of the thresholding methodology are presented elsewhere (18). This task did not show a difference between controls and participants with VS (17).

### Transcranial Magnetic Stimulation: Determining Phosphene Threshold

As described by Stewart and colleagues (19), the phosphene threshold is used to determine the personalized “dose” of TMS that will be used for each subject. The phosphene threshold is the “dose” of TMS that is necessary to result in the perception of phosphenes as described. For this study, this is done before the first TMS treatment and determined again at the start of week 2 (or treatment session 6) of the 10-session treatment schedule. To determine the phosphene threshold, participants wear a blindfold and a cap is worn on the head. Three points positioned over the occipital midline and 2, 3, and 4 cm above the inion are marked. The TMS coil is positioned such that the handle points upwards and is parallel to the subject's spine. Single pulse TMS is then applied over one of the marked points and the subject reports the presence or absence of a phosphene immediately after stimulation. Stimulation is initially applied at 60% of stimulator output. If the subject reliably perceives a phosphene, reporting it five or more times out of ten, intensity is reduced in steps of 5% and stimulation will be again given ten times. Stimulation intensity is reduced until the subject no longer reliably perceives a phosphene. Stimulation intensity is then increased in blocks of 5% until the minimum intensity at which the subject can perceive a phosphene five times out of 10 is established and this value is determined to be the threshold. If the participant does not initially perceive a phosphene at 60% of stimulator output, intensity is increased in blocks of 5% to a maximum level of 100% of stimulator output. If the subject fails to perceive a phosphene at the maximum level, the coil position is shifted to another of the points marked on the cap and the procedure will be repeated until the threshold is determined at one of the marked points.

### Transcranial Magnetic Stimulation: Treatment Procedure

For treatment in this pilot study, bilateral low-frequency (1 Hz) repetitive transcranial magnetic stimulation (LF-rTMS) is administered to both right and left lingual gyri using a custom-built, 120°-angulated, 80 mm double figure-of-eight coil manufactured by Magstim Ltd (Whitland, Camarthenshire, UK). Targets are selected based on visual inspection of a participant's T1-weighted MRI images of the brain, and individual target coordinates are recorded in Montreal Neurological Institute (MNI) space. The TMS coil is positioned over the specified target location based upon the participant's MRI image in MNI space facilitated by the Brainsight™ interface, and LF-rTMS is administered at 110% of the phosphene threshold using the determined target and trajectory. Treatment sessions include two 15–20 min trains (one train per side) for a total of no more than 1,800 stimulations during each treatment session. Each session includes a total of 30–40 min of LF-rTMS stimulation time with a brief break in between sides. For each session, after treatment is complete, the participant is given a side effect survey. The rTMS treatment sessions occur daily for 5 days per week for two consecutive weeks.

### Data Analysis

#### Side Effects

To determine whether any participant experiences untoward effects of TMS, a side effect survey is being used, as noted, and summary reports of adverse events will be published. It should be known that the cortical location of the treatment for this trial is different from previous studies, so it is possible to encounter side-effects not previously reported.

#### Drop-Out Rate Estimates

With a sample size of 10, we will be able to estimate the expected drop-out rate for larger studies in the range of 20–50% to within a 95% confidence interval of ±25% to ±31%. To calculate a dropout rate for future studies and shrink the confidence interval, we will use data gathered in this study and data from chronic tinnitus studies using TMS to perform a Bayesian analysis for drop-out estimation. For reference, the available studies [see (12)] for tinnitus and rTMS revealed an approximate dropout rate of 7% in rTMS group and 12% in the sham group.

#### Performance of Outcome Measures

To define the performance of the CVSS and the performance of the three psychophysical visual processing tasks, the standard deviation and test-retest reliability for CVSS and each of the three psychophysical tasks will be determined. The CVSS and the three psychophysical tasks will be given pre-treatment and then repeated on the 1st day of treatment prior to rTMS, and these results will be used to conclude the test-retest reliability using the intraclass correlation coefficient (with a two-way mixed effects, absolute agreement, single rater/measurement model).

#### Outcome Measure Changes With Treatment

Changes in outcome measures (i.e., changes in the CVSS, VFQ-25, and GAD-7) with treatment will be assessed. Results pre-treatment (first day of treatment prior to rTMS) and results after last day of treatment with rTMS will be compared by assessing within-subject correlations (i.e., repeated measures correlation) for the CVSS, VFQ-25, GAD-7, and the suppression index (center-surround task), detection threshold (luminance detection task), and coherence threshold (global motion task). The effect size for each measure using a linear mixed model will be determined.

## Risks and Protection Against Risks

All of the parameters proposed for this study fall within the accepted parameters for safe rTMS administration with an estimated risk of <1 in 10,000 of inducing seizures in appropriately screened subjects (14–16). Overall, low frequency-rTMS protocols, such as the one used in this pilot study, are considered to be of minimal risk for serious adverse events and have been used extensively in previous research (20). LF-rTMS has been applied to over a hundred subjects with other cortical hyperexcitability syndromes (largely tinnitus and central pain) with no reports of seizures or other serious adverse events (21–23). A slight risk of headache and neck pain is expected, but these symptoms are typically self-resolving and/or treatable with over-the-counter analgesics. Other potential side effects include scalp discomfort at the site of stimulation, scalp or jaw or face tingling or muscle spasms, light headedness, and visual blurring.

To mitigate risks, all participants are screened prior to TMS (see exclusion criteria). Each participant is required to have a MRI scan performed within 3 years that does not show any concerning lesion. There have been reports of hearing loss with repeated TMS pulses, and thus all participants and investigators are required to wear ear plugs, consistent with what is worn during an MRI brain scan. An on-call neurologist is available at all times in the event of a seizure or other adverse study event. Study personnel involved in human subject interactions are BLS certified and specifically trained in seizure safety and what to do in the event of other medical emergencies. All adverse events are reported to the Colorado Multiple Institutional Review Board (COMIRB), and if a seizure were to occur, it would also be reported to the FDA. To monitor for adverse events, and make appropriate modifications, a side effects survey is given to each participant after each treatment session and at month one and month three after treatment is completed.

## Discussion

Only one participant completed the study before the COVID-19 pandemic restricted all studies on campus. This section will focus on side effects noted, the approach to the challenges encountered due to the COVID-19 pandemic, and lessons learned thus far that will inform the remainder of the trial and future treatment trials of VS using rTMS.

### Side Effects

During the rTMS sessions, the participant who completed the study experienced symptoms consistent with twitching of the face and scalp, and the feeling of a tapping sensation on the skull, which are common during TMS procedures. In one instance, while targeting the right lingual gyrus, the contralateral upper shoulder/lower neck region would twitch in unison with each pulse. This occurred for less than a few minutes into one session and was reported as uncomfortable, but not painful, and resolved after slight adjustment of the TMS coil. It is worth noting that this participant had a phosphene threshold of 87% and, therefore, a relatively high stimulator output of 96% for dosage. After several daily sessions, the participant also reported mild light headedness, and very mild blurred vision and tingling in the hands. Based on further discussion, it is possible these side effects arose from the position the participant was placed in during the sessions, as symptoms resolved with alteration of the participant's position on one occasion. During TMS, each participant is seated, with their heads facing down on a pillow and their arms resting with hands together or side-by-side above the head. It is not possible to rule outside effects due to TMS given the nature of the symptoms, but symptoms were confirmed to be resolved before the end of each visit where the symptoms were reported.

### Implementation of Psychophysical Visual Processing Tasks

A similar experimental model to that used by McKendrick et al. in their 2017 study on behavioral measures of cortical hyperexcitability was implemented for this pilot study and was adapted to an application downloaded onto a tablet (17, 18). Several test trials ensured that the tasks and application were working and that all anonymized data were instantly uploaded to a cloud-based server, which has made data sharing streamed-lined and effortless. Outside of a few minor operating system issues, implementation and completion of visual processing tasks with the participant enrolled went smoothly. For future studies, this process is desirable in order to have all data processed in a blinded fashion at one center.

### The Impact of COVID-19 Pandemic

The pilot study was halted before opening for enrollment due to the COVID-19 pandemic in March 2020. Once the campus was open for in-person and on-campus treatment trials in the late Summer of 2020, additional documentation and processes were necessary before the pilot study was approved by campus research officials for recruitment. A required COVID-19 mitigation plan was submitted and included a designated COVID-19 officer for the TMS laboratory space, a plan to follow all campus and CDC guidelines with proper cleaning procedures, use of personal protective equipment, screening questionnaires for exposure to, and symptoms of, COVID-19 for participants, and a controlled check-in location for screening and temperature checks. In addition, a high-efficiency particulate air filter was purchased for use during TMS sessions to ensure the safety of the TMS technician and participants, due to the proximity (<6 feet) required during TMS treatments. Despite these measures and modifications, all but one of the potential participants who were previously screened and deemed eligible decided not to participate throughout 2020 and into the Spring of 2021. Although many potential participants contacted for the study continue to have concerns about onsite visits and travel to the site daily for 2 weeks during the pandemic, as the risks for COVID-19 are better understood and as the pandemic is under better control with increasing vaccination rates, additional eligible participants are now in the process of scheduling sessions. An unexpected consequence of these delays due to the COVID-19 pandemic is the fact that brain MRI scans are no longer falling within the 3-year cut-off time point for potential participants who were determined to be eligible before the pandemic began.

### Other Recruitment Challenges

The initial review of the electronic medical record allowed for the identification of those diagnosed with VS syndrome. Only 34% of those identified with a diagnosis of VS were eligible based on review of records. Using data regarding the safety of rTMS in all subjects, the upper limit of age eligibility that was initially proposed was increased from 40 to 65 years and this increased the pool of potential participants by 20%. Approximately 14% of those initially identified by diagnostic codes were ineligible because their brain MRI scan had been performed >3 years prior, and that number grew after delays due to the pandemic. Approximately 62% of contact attempts via phone to those deemed eligible by record review were successful. The most common factor that determined whether the potential participant had continued interest in the study, after initial contact, was the subjective degree of impact of the VS syndrome on their daily activities. Those who reported that daily activities were significantly impacted by VS were more likely to be willing to consider ways to participate in the future and alter their daily routines and work schedules to be available for study visits. Beyond concerns related to the COVID-19 pandemic, the most common reasons for potential participants to decline to take part in the study was disruption to work schedules and personal obligations, followed by duration of travel to and from the study site. Due to the relative rarity of the VS syndrome, many of the potential participants that were contacted live out of the immediate area or live out of state. Those with commutes over 30 min were the least likely to ask to be called back after the COVID-19 pandemic was under better control and vaccinations were more common.

### Challenges and Modifications to the TMS Schedule

Numerous published studies indicate rTMS is more likely to be effective if performed in succession over multiple days for two or more weeks. Thus, the initial schedule proposed in the protocol included consecutive sessions, 5 days a week for 2 weeks. With the first participant, unforeseen circumstances related to personal and work obligations made it apparent that in order for this pilot study to be successful, one missed session per week should be allowed. For the remainder of the study, flexibility will be maintained in this manner. Another modification that increased interest in the study and interest future contact for participation (for those not comfortable participating during the pandemic) was the ability to schedule study visits in the early morning, late afternoon, or early evening.

### Summary

Currently there are no effective treatments for the VS syndrome, which converging lines of evidence suggest may be a hyperexcitability syndrome. This open-label treatment trial of rTMS for VS syndrome is ongoing, and results will be used to inform the feasibility and utility of a future randomized, controlled trial of rTMS for VS syndrome. The greatest challenge faced in the ongoing study has been difficulty with recruitment during to the COVID-19 pandemic. However, with decreasing COVID-19 restrictions within the United States and the increase in COVID-19 vaccinations in Colorado, there is renewed interest in participation in the study by those previously screened. Given the recent progress to date, the current aim is to complete enrollment by June 2022. Following completion of the study and data analyses, feasibility for future studies will be determined. Ultimately, due to the rarity of the VS syndrome, and the potential under-diagnosis of VS, a multicenter treatment trial will most likely be needed to recruit enough participants to assess treatment efficacy.

Although the challenges faced in this pilot study to date have been, by and large, related to the COVID-19 pandemic, the lessons learned also provide insights for future treatment trials. For example, consecutive daily visits disrupt schedules to a greater degree than a similar number of visits over a greater period of time. Trials in the future should include reimbursement to participants that commensurates to the burden of the schedule, which is greater than the usual for a similar number of visits over a greater period of time in other types of treatment trials, such as pharmaceutical interventions. The budget should also include reimbursement for travel, hotel costs, and, in some instances, airline travel. These measures would help relieve the burden of concentrated visits for TMS and help address the recruitment issues associated with a rare condition. Finally, budgeting for standard brain MRI scans for those with scans more than 3 years prior to enrollment should also improve eligibility and enrollment.

## Ethics Statement

Approval for this study was obtained through the Colorado Multiple Institutional Review Board (COMIRB) and written informed consent was obtained, and will be obtained, for all participants. Results will be published following completion of the study and data analyses.

## Author Contributions

VP and IB contributed to conception and design of the study. MG and LL carried out study activities and assisted with protocol amendments. MG and VP wrote the first draft of the manuscript. LL contributed to sections of the manuscript and specifically sections on conducting TMS. All authors contributed to manuscript revision, read, and approved the submitted version.

## Funding

The Treatment Trial was sponsored by the Visual Snow Initiative, which is a public charity under the fiscal sponsorship of the Edward Charles Foundation, a non-profit 501(c)(3) organization, EIN# 26-4245043.

## Conflict of Interest

The authors declare that the research was conducted in the absence of any commercial or financial relationships that could be construed as a potential conflict of interest.

## Publisher's Note

All claims expressed in this article are solely those of the authors and do not necessarily represent those of their affiliated organizations, or those of the publisher, the editors and the reviewers. Any product that may be evaluated in this article, or claim that may be made by its manufacturer, is not guaranteed or endorsed by the publisher.
