# Supplementary material for: A Study Protocol for an Open-Label Feasibility Treatment Trial of Visual Snow Syndrome With Transcranial Magnetic Stimulation
Source: Front Neurol. 2021 Sep 24;12:724081. doi: 10.3389/fneur.2021.724081 (PMC8500216; doi:10.3389/fneur.2021.724081)
Supplement: Supplementary file 1 [file Data_Sheet_1.docx]

Colorado Visual Snow Scale

Instructions: Give at Visits 1, 2, 11, 12, and 13

Visit 1: Answer questions for experience during past 1 month pre-treatment

Visit 2: Answer questions for experience during past 1 month pre-treatment

Visit 11: Answer questions for past 2 days (this will be last day of TMS)

Visit 12: Answer questions for experience during past 1 month post-treatment

Visit 13: Answer questions for experience during past 2 months (i.e. since Visit 11)

| 0 | 1 | 2 | 3 | 4 | 5 | 6 | 7 | 8 | 9 | 10 |
| --- | --- | --- | --- | --- | --- | --- | --- | --- | --- | --- |
| None or  Not Felt | Mild/  Weak | | | Moderate/  Strong | | | Severe/  Extreme | | | Worse Ever |

1. Visual Static

1. The visual intensity of the visual static is:

0 1 2 3 4 5 6 7 8 9 10

1. The degree that the visual static interferes with my ability to see:

0 1 2 3 4 5 6 7 8 9 10

1. The degree that the visual static interferes with my ability to function in daily activities related to work or school (if not working or in school, then daily activities):

0 1 2 3 4 5 6 7 8 9 10

1. The degree that the visual static interferes with my ability to drive:

0 1 2 3 4 5 6 7 8 9 10

2. Afterimages

1. The visual intensity of the afterimages is:

0 1 2 3 4 5 6 7 8 9 10

2. The degree that the afterimages interfere with my ability to see:

0 1 2 3 4 5 6 7 8 9 10

3. The degree that the afterimages interfere with my ability to function in daily activities related to work or school (if not working or in school, then daily activities):

1. 1 2 3 4 5 6 7 8 9 10

4.iiiiiiiThe degree that the afterimages interfere with my ability to drive:

0 1 2 3 4 5 6 7 8 9 10

3. Trails

1. The intensity of the trails is:

0 1 2 3 4 5 6 7 8 9 10

2. The degree that the trails interfere with my ability to see:

0 1 2 3 4 5 6 7 8 9 10

3. The degree that the trails interfere with my ability to function in daily activities related to work or school (if not working or in school, then daily activities):

1. 1 2 3 4 5 6 7 8 9 10

4.iiiiiiiThe degree that the trails interfere with my ability to drive:

0 1 2 3 4 5 6 7 8 9 10

4. Blue field entoptic phenomenon

1. The severity of the blue field entoptic phenomenon is:

0 1 2 3 4 5 6 7 8 9 10

2. The degree that the blue field entoptic phenomenon interferes with my ability to see:

0 1 2 3 4 5 6 7 8 9 10

3. The degree that the blue field entoptic phenomenon interferes with my ability to function in daily activities related to work or school (if not working or in school, then daily activities):

1. 1 2 3 4 5 6 7 8 9 10

4.iiiiiiiThe degree that the blue field entoptic phenomenon interferes with my ability to drive:

0 1 2 3 4 5 6 7 8 9 10

5. Floaters

1. The severity of the floaters is:

0 1 2 3 4 5 6 7 8 9 10

2. The degree that the floaters interfere with my ability to see:

0 1 2 3 4 5 6 7 8 9 10

3. The degree that the floaters interfere with my ability to function in daily activities related to work or school (if not working or in school, then daily activities):

0 1 2 3 4 5 6 7 8 9 10

4. The degree that the floaters interfere with my ability to drive:

0 1 2 3 4 5 6 7 8 9 10

6. Night vision problems

1. The severity of my night vision problems is:

0 1 2 3 4 5 6 7 8 9 10

2. The degree that my night vision problems interfere with my ability to see:

0 1 2 3 4 5 6 7 8 9 10

3. The degree that my night vision problems interfere with my ability to function in daily activities related to work or school (if not working or in school, then daily activities):

0 1 2 3 4 5 6 7 8 9 10

4.iiiiiiiThe degree that my night vision problems interfere with my ability to drive:

0 1 2 3 4 5 6 7 8 9 10

7. Ringing in the ears (Tinnitus) – if never experienced, skip

1. The severity of the ringing in my ears is:

0 1 2 3 4 5 6 7 8 9 10

2. The degree that the ringing in my ears interferes with my ability to see:

0 1 2 3 4 5 6 7 8 9 10

3. The degree that that the ringing in my ears interferes with my ability to function in daily activities related to work or school (if not working or in school, then daily activities):

1. 1 2 3 4 5 6 7 8 9 10

4.iiiiiiiiThe degree that that the ringing in my ears interferes with my ability to drive:

0 1 2 3 4 5 6 7 8 9 10

8. The feeling of detachment – if never experienced, skip

1. The severity of feelings of detachment is:

0 1 2 3 4 5 6 7 8 9 10

2. The degree that the feelings of detachment interfere with my ability to see:

0 1 2 3 4 5 6 7 8 9 10

3. The degree that that the feelings of detachment interfere with my ability to function in daily activities related to work or school (if not working or in school, then daily activities):

1. 1 2 3 4 5 6 7 8 9 10

4. iiiiiiThe degree that that the feelings of detachment interfere with my ability to drive:

0 1 2 3 4 5 6 7 8 9 10

9. Anxiety

1. The severity of anxiety is:

0 1 2 3 4 5 6 7 8 9 10

2. The degree that anxiety interferes with my ability to see:

0 1 2 3 4 5 6 7 8 9 10

3. The degree that anxiety interferes with my ability to function in daily activities related to work or school (if not working or in school, then daily activities):

0 1 2 3 4 5 6 7 8 9 10

1. The degree that anxiety interferes with my ability to drive:

0 1 2 3 4 5 6 7 8 9 10

10. Depression

1. The severity of depression is:

0 1 2 3 4 5 6 7 8 9 10

2. The degree that depression interferes with my ability to see:

0 1 2 3 4 5 6 7 8 9 10

3. The degree that depression interferes with my ability to function in daily activities related to work or school (if not working or in school, then daily activities):

0 1 2 3 4 5 6 7 8 9 10

4. The degree that depression interferes with my ability to drive:

0 1 2 3 4 5 6 7 8 9 10

11. Sleep issues

1. The severity of my sleep issue is:

0 1 2 3 4 5 6 7 8 9 10

2. The degree that my sleep issues interfere with my ability to see:

0 1 2 3 4 5 6 7 8 9 10

3. The degree that my sleep issues interfere with my ability to function in daily activities related to work or school (if not working or in school, then daily activities):

0 1 2 3 4 5 6 7 8 9 10

4. The degree that my sleep issues interfere with my ability to drive:

0 1 2 3 4 5 6 7 8 9 10

Additional question at Visit 11: Please list any new symptoms (positive or negative) that you have experienced since the TMS:

Additional question at Visit 12: Please list any new symptoms (positive or negative) that you have experienced since your last visit 1 month ago:

Additional question at Visit 13: Please list any new symptoms (positive or negative) since your last visit 2 months ago:
